# Neuroprognostication After Extracorporeal Cardiopulmonary Resuscitation: ECMO-Specific Challenges and a Multimodal Time-Sensitive Framework

**DOI:** 10.3390/jcdd13080364

**Published:** 2026-08-02

**Authors:** Debora Emanuela Torre, Carmelo Pirri

**Affiliations:** 1 Department of Cardiac Anesthesia and Intensive Care Unit, Cardiac Surgery, Ospedale dell’Angelo, Mestre, 30174 Venice, Italy; deboraemanuela.torre@aulss3.veneto.it; 2 Department of Neurosciences, Institute of Human Anatomy, University of Padova, 35121 Padua, Italy

**Keywords:** neuroprognostication, extracorporeal cardiopulmonary resuscitation, post-cardiac arrest care, neurological outcome, cardiac arrest

## Abstract

Extracorporeal cardiopulmonary resuscitation (ECPR) has emerged as a promising strategy for selected patients with refractory cardiac arrest, improving survival and the likelihood of favorable neurological outcomes. However, neurological prognostication in this setting remains highly challenging and insufficiently standardized. The pathophysiological complexity of ECPR, including global ischemia–reperfusion injury, altered cerebral perfusion, systemic inflammation, anticoagulation and prolonged sedation, limits the reliability of conventional post-cardiac arrest prognostic tools. This narrative review provides a focused and clinically oriented synthesis of current evidence on brain injury and neuroprognostication in patients undergoing veno-arterial extracorporeal membrane oxygenation (V-A ECMO) for cardiac arrest. Key determinants of neurological outcome across pre-ECMO and peri-resuscitation phases are examined, alongside the role and limitations of multimodal monitoring strategies, including neurological examination, electroencephalography, neuroimaging, cerebral oximetry and circulating biomarkers. Particular attention is given to the timing of prognostication and the risk of premature or inaccurate predictions leading to self-fulfilling prophecies. Emerging data suggest that neurological recovery in ECPR patients may be delayed, supporting a more cautious and time-adapted approach. A pragmatic, multimodal framework for neurological assessment in this population is outlined. By addressing current gaps and proposing a structured approach, this review aims to inform clinical decision making and contribute to improved neurologically meaningful survival in ECPR-treated cardiac arrest.

## 1. Introduction

Extracorporeal cardiopulmonary resuscitation (ECPR) has progressively emerged as a rescue strategy for selected patients with refractory cardiac arrest, particularly in high-volume centers with established extracorporeal membrane oxygenation (ECMO) programs [1,2]. By restoring systemic perfusion when conventional cardiopulmonary resuscitation fails, ECPR has been associated with improved survival and, in a subset of patients, favorable neurological outcomes [3,4]. Nevertheless, neurological injury remains the leading determinant of long-term prognosis, and its assessment represents one of the most complex and unresolved challenges in contemporary critical care [5]. Neuroprognostication after cardiac arrest has traditionally relied on a combination of clinical examination, electrophysiological studies, neuroimaging and circulating biomarkers, applied within relatively well-defined temporal windows [5]. However, the application of these paradigms to patients undergoing ECPR is fraught with limitations [6]. The pathophysiological milieu of ECPR differs substantially from that of conventional post-cardiac arrest care, as it combines the effects of prolonged low-flow states with the non-physiological hemodynamics of extracorporeal circulation. In addition, deep sedation, neuromuscular blockade, systemic inflammation, anticoagulation and metabolic derangements further obscure neurological assessment and may profoundly alter the performance of established prognostic tools [7,8]. As a consequence, both the timing and the reliability of neurological evaluation are significantly compromised. These limitations may lead to inaccurate early prognostic assessment and support a more cautious interpretation of neurological findings in ECPR-treated patients [5]. Despite the growing use of ECPR, evidence specifically addressing neuroprognostication in this setting remains limited and is largely extrapolated from conventional cardiac arrest populations, with uncertain applicability [9,10,11]. This gap highlights the need for an ECPR-specific framework that integrates pathophysiological insights with a cautious, multimodal and time-adapted clinical approach. This narrative review aims to provide a clinically oriented synthesis of current evidence on brain injury and neurological assessment in patients undergoing veno-arterial ECMO for cardiac arrest, highlighting both the potential and the limitations of available tools and proposing a pragmatic approach to guide clinical decision making. Rather than proposing novel prognostic markers, the aim of this review is to examine how ECPR-specific physiological and clinical factors modify the interpretation, reliability and timing of established neuroprognostic modalities. Importantly, this review is not intended to provide formal clinical recommendations or a validated prognostic algorithm. Rather, it aims to summarize current evidence and propose a conceptual framework to support multimodal neuroprognostication in a field where ECPR-specific evidence remains limited.

## 2. Materials and Methods

This narrative review was conducted to provide a clinically oriented and pathophysiologically grounded synthesis of current evidence on brain injury and neuroprognostication in patients undergoing extracorporeal cardiopulmonary resuscitation (ECPR) supported with veno-arterial extracorporeal membrane oxygenation (V-A ECMO). A comprehensive literature search was performed using major electronic databases including PubMED, Scopus and Embase, focusing on studies published up to April 2026. The search strategy combined relevant keywords and medical subject headings (MeSH) terms related to cardiac arrest and extracorporeal support, including “ECPR”, “extracorporeal cardiopulmonary resuscitation”, “V-A ECMO”, “cardiac arrest”, “neuroprognostication”, “neurological outcome”, and “brain injury”. Additional articles were identified through the manual screening of reference lists of selected studies and relevant reviews. Studies primarily focused on ECPR, cardiac arrest, V-A ECMO, neurological injury, neuroprognostication or brain death determination were preferentially included. Studies involving broader V-A ECMO populations were also considered when they provided clinically relevant information regarding cerebral physiology, neuromonitoring, neurological injury or prognostic assessment applicable to ECPR patients. Studies exclusively focused on pediatric populations, non-cardiac extracorporeal support modalities or topics unrelated to neurological assessment were generally excluded unless they provided important mechanistic insights relevant to ECPR. Given the narrative nature of this review, no formal systematic selection process or meta-analytic techniques were applied. Although a structured literature search was performed across multiple databases, article selection was primarily guided by clinical relevance, methodological quality and applicability to neuroprognostication in ECPR. Therefore, no formal PRISMA flow diagram or quantitative reporting of study selection was undertaken. Overall, approximately 123 references were included in the final narrative synthesis. Instead, studies were selected based on their relevance to the topic, methodological quality and clinical applicability. Priority was given to original research articles, prospective and retrospective cohort studies and high-quality reviews addressing neurological outcomes and prognostic assessment in cardiac arrest and ECMO populations.

When multiple publications addressed similar topics, preference was given to more recent studies, larger cohorts, prospective investigations and articles specifically evaluating ECPR populations. International guidelines and consensus statements were also included when relevant to clinical neuroprognostication.

When evidence specific to ECPR was limited, data from conventional post-cardiac arrest populations were considered, with critical appraisal of their applicability to the ECMO setting. Particular attention was devoted to studies exploring the pathophysiology of hypoxic–ischemic brain injury, the impact of extracorporeal circulation on cerebral hemodynamics and the performance and limitations of multimodal prognostic tools, including clinical examination, electroencephalography, neuroimaging, cerebral oximetry and circulating biomarkers. Particular attention was devoted to studies reporting neurological outcomes after ECPR, which were summarized in a dedicated evidence table. Emerging concepts such as delayed neurological recovery and ECMO-specific confounders were also specifically investigated. The available evidence was narratively synthesized with particular focus on ECMO-specific pathophysiology, neuroprognostication strategies and clinically relevant challenges in neurological assessment. No formal risk-of-bias assessment or evidence grading was performed; the conclusions should therefore be interpreted within the context of a narrative synthesis rather than a systematic evidence review.

## 3. Narrative Synthesis of Current Evidence

### 3.1. Pathophysiology of Brain Injury in ECPR

Brain injury in patients undergoing extracorporeal cardiopulmonary resuscitation is the result of a complex and dynamic interplay between primary ischemic insult and secondary injury mechanisms occurring during and after the initiation of extracorporeal support (Table 1) [5]. Unlike conventional cardiac arrest, ECPR introduces additional pathophysiological layers that profoundly influence both the extent and the evolution of cerebral damage. From a conceptual perspective, brain injury in this setting may be broadly categorized into primary and secondary components [5,12]. The primary injury is mainly determined by the duration and quality of no-flow and low-flow states preceding the establishment of extracorporeal circulation, leading to global cerebral ischemia and energy failure. Secondary injury develops during reperfusion and ongoing extracorporeal support and is driven by a combination of systemic and cerebral factors, including oxidative stress, neuroinflammation and alterations in cerebral perfusion [5]. A distinctive feature of ECPR is the presence of non-physiological hemodynamics associated with veno-arterial ECMO, which may impair cerebrovascular autoregulation and contribute to heterogeneous cerebral blood flow. In addition, extracorporeal support may expose the brain to regionally variable oxygenation, particularly in the context of differential hypoxia [13]. Overall, brain injury in ECPR is characterized by marked temporal and spatial heterogeneity, reflecting the interaction between pre-resuscitation factors, reperfusion injury and ECMO-related alterations in cerebral physiology. This complexity has important implications for neuroprognostication and underscores the need for a structured, multimodal and time-sensitive approach.

#### 3.1.1. Global Ischemia–Reperfusion Injury

Global cerebral ischemia–reperfusion injury represents the central and most extensively studied mechanism of brain damage in patients undergoing extracorporeal cardiopulmonary resuscitation. It originates from the combined effects of no-flow and low-flow states preceding the initiation of extracorporeal support, followed by the complex cascade triggered by reperfusion [5,7,12]. During the no-flow phase, the complete interruption of cerebral blood flow leads to rapid depletion of high-energy phosphates, loss of membrane ionic gradients and failure of adenosine triphosphate-dependent cellular processes. This results in neuronal depolarization; excessive release of excitatory neurotransmitters, particularly glutamate; and subsequent activation of N-methyl-D-aspartate receptors, promoting calcium influx and excitotoxic injury. Even short periods of untreated cardiac arrest can initiate irreversible neuronal damage, particularly in highly vulnerable regions such as the hippocampus and cerebral cortex [14,15]. The subsequent low-flow phase, typically associated with suboptimal cardiopulmonary resuscitation, provides insufficient cerebral perfusion to meet metabolic demands, thereby exacerbating ischemic injury while partially delaying complete cellular necrosis. The duration and quality of this phase are critical determinants of the extent of primary brain injury and are strongly associated with neurological outcomes [5,16,17]. Reperfusion, although essential to tissue survival, paradoxically contributes to further neuronal damage through a series of interconnected mechanisms. The abrupt restoration of oxygen delivery leads to the generation of reactive oxygen species, mitochondrial dysfunction and oxidative stress. In parallel, intracellular calcium overload and activation of proteolytic and lipolytic enzymes amplify cellular injury. These processes are further aggravated by endothelial dysfunction and disruption of the blood–brain barrier, resulting in vasogenic edema and increased intracranial pressure [18,19]. In the context of ECPR, reperfusion injury may be particularly pronounced due to the often prolonged duration of low-flow states prior to ECMO initiation. Moreover, the rapid restoration of systemic circulation by extracorporeal support may not translate into homogeneous microvascular reperfusion, thereby perpetuating cellular hypoxia at the tissue level despite apparently adequate microcirculatory parameters [5,7,20]. From a clinical perspective, global ischemia–reperfusion injury represents the primary substrate upon which subsequent ECMO-related factors act, shaping the overall trajectory of brain damage. Its severity largely determines the baseline probability of neurological recovery and significantly influences the performance and interpretation of prognostic tools [5]. Importantly, the temporal evolution of ischemia–reperfusion injury is highly dynamic, with secondary processes unfolding over hours to days, further complicating early neuroprognostication.

#### 3.1.2. ECMO-Related Cerebral Hemodynamics

V-A ECMO profoundly alters systemic and cerebral hemodynamics, introducing non-physiological flow patterns that may significantly impact cerebral perfusion and contribute to brain injury. Unlike native cardiac output, which is characterized by pulsatile flow and finely regulated autoregulatory mechanisms, ECMO support often generates continuous or minimally pulsatile flow, thereby modifying the normal coupling between systemic pressure and cerebral blood flow [8,21,22,23]. Under physiological conditions, cerebral autoregulation maintains relatively stable cerebral blood flow across a wide range of mean arterial pressures [24]. However, in patients undergoing ECPR, this mechanism may be impaired as a result of global ischemia, endothelial dysfunction and systemic inflammation [25]. The combination of disrupted autoregulation and non-pulsatile ECMO flow may render cerebral perfusion increasingly pressure-dependent, exposing the brain to both hypoperfusion and hyperperfusion depending on systemic hemodynamic conditions [25,26]. In this context, the relationship between mean arterial pressure and cerebral flow becomes highly variable and difficult to predict. Excessively low perfusion pressures may exacerbate ongoing ischemia, while excessively high pressures may contribute to cerebral edema and increase the risk of hemorrhagic transformation [27,28]. This delicate balance is further complicated by the frequent use of vasopressors and inotropes, which may have heterogeneous effects on cerebral circulation [29]. Carbon dioxide tension represents another key determinant of cerebral hemodynamics in ECMO-supported patients. PaCO_2_ is a potent modulator of cerebrovascular tone, with hypercapnia inducing vasodilation and increased cerebral blood flow and hypocapnia leading to vasoconstriction and reduced perfusion [30]. Rapid fluctuations in PaCO_2_, which may occur during initiation or adjustment of ECMO support, can therefore result in abrupt changes in cerebral blood flow, potentially exacerbating neuronal injury [31,32]. Oxygenation dynamics also play a critical role. While ECMO provides efficient systemic oxygen delivery, arterial oxygen tension may vary depending on the balance between native cardiac output and extracorporeal flow [33]. Both hypoxemia and hyperoxia have been associated with adverse neurological outcomes, the latter through mechanisms involving oxidative stress and vasoconstriction. Thus, maintaining an appropriate oxygenation range is essential to minimizing secondary brain injury [34,35]. Another distinctive feature of ECMO-related hemodynamics is the interaction between native cardiac function and extracorporeal flow [33]. In peripheral V-A ECMO, retrograde flow from the arterial cannula competes with antegrade flow from the recovering left ventricle, creating a variable “mixing zone” within the aorta. The position of this mixing zone determines the oxygenation and perfusion of cerebral circulation and may shift dynamically over time, further contributing to variability in cerebral oxygen delivery [36]. Overall, ECMO-related alterations in cerebral hemodynamics introduce significant spatial and temporal variability in brain perfusion, challenging the assumption of stable and homogenous cerebral blood flow. These factors not only contribute to ongoing brain injury but also complicate the interpretation of clinical and instrumental markers of neurological function, thereby representing a major limitation for reliable neuroprognostication in ECPR-treated patients.

#### 3.1.3. Neuroinflammation and Coagulation Disorders

Neuroinflammation and coagulation abnormalities represent key contributors to secondary brain injury in patients undergoing extracorporeal cardiopulmonary resuscitation, reflecting the complex interplay between ischemia–reperfusion injury, systemic inflammatory response and extracorporeal circulation [5,37,38]. Following global cerebral ischemia, reperfusion triggers a robust neuroinflammatory cascade characterized by activation of microglia, astrocytes and endothelial cells. This response is mediated by the release of pro-inflammatory cytokines, chemokines and damage-associated molecular patterns, which amplify local and systemic inflammation [5,39,40,41]. A central consequence of this inflammatory response is the disruption of the blood–brain barrier [42]. Increased permeability of the blood–brain barrier facilitates the extravasation of plasma proteins and inflammatory cells into the cerebral parenchyma, contributing to vasogenic edema and increased intracranial pressure [43]. This process not only worsens neuronal injury but also creates a permissive environment for further secondary damage, including microvascular dysfunction and impaired oxygen delivery. Extracorporeal circulation itself plays a significant role in amplifying systemic inflammation. Contact between blood components and the artificial surfaces of the ECMO circuit activates complement pathways, leukocytes and platelets, leading to a systemic inflammatory response syndrome that may persist throughout the duration of support. This ECMO-related inflammation may further aggravate cerebral injury by promoting endothelial activation, capillary leakage and microcirculatory impairment [5,44]. Coagulation disorders are closely intertwined with inflammatory processes and represent another clinical determinant of neurological complications in ECPR patients. The simultaneous activation of procoagulant and anticoagulation pathways may result in a fragile hemostatic balance, characterized by a high risk of both thrombotic and hemorrhagic events [44,45]. On one hand, formation of microthrombi within the cerebral microcirculation may exacerbate ischemic injury by impairing capillary perfusion. On the other hand, systemic anticoagulation, required to maintain circuit patency, combined with platelet dysfunction and coagulopathy increases the risk of intracranial hemorrhage [46,47]. This dual vulnerability, encompassing both ischemic and hemorrhagic mechanisms, contributes to the heterogeneous pattern of brain injury observed in ECPR-treated patients. Importantly, the coexistence of these processes may complicate neuroimaging interpretation and further limit the predictive value of individual prognostic markers. From a prognostic perspective, the dynamic interaction between neuroinflammation and coagulation abnormalities introduces a substantial variability in the evolution of brain injury over time. These processes may evolve over hours to days following ECMO initiation, thereby reducing the reliability of early prognostic assessment and reinforcing the need for a multimodal and longitudinal approach to neuroprognostication [6,10].

#### 3.1.4. Sedation and Metabolic Confounders

Sedation and metabolic disturbances represent major confounding factors in the assessment of neurological function in patients undergoing extracorporeal cardiopulmonary resuscitation, significantly limiting the reliability and interpretability of clinical and instrumental findings [8,10]. Deep sedation is frequently required in patients supported with V-A ECMO to ensure ventilatory synchrony, prevent patient–device interactions and maintain hemodynamic stability. However, the pharmacokinetics and pharmacodynamics of sedative and analgesic agents are profoundly altered in this setting. Hemodilution, drug sequestration within extracorporeal circuit and variable organ perfusion, particularly hepatic and renal, contribute to unpredictable drug distribution, delayed clearance and potential accumulation. As a consequence, prolonged depression of consciousness may persist even after discontinuation of sedative agents, making early neurological examination unreliable [48,49,50]. Neuromuscular blocking agents, often used in the initial phases of ECPR, further obscure clinical assessment by abolishing motor responses, thereby precluding the evaluation of one of the key components of neurological examination. In addition, residual neuromuscular blockade may persist beyond the expected duration, particularly in the presence of organ dysfunction, leading to potential misinterpretation of neurological status [51,52,53]. Metabolic derangements are highly prevalent in this population and may independently affect cerebral function. Hypoxia, hypercapnia or hypocapnia, acid–base imbalances, electrolyte disturbances and glycemic variability can all influence neuronal activity and level of consciousness [8,32,53]. In particular, alterations in carbon dioxide tension may significantly modulate cerebral flow, while severe metabolic acidosis or alkalosis may impair neuronal excitability and synaptic transmission. Furthermore, systemic shock and multiorgan failure, which frequently accompany refractory cardiac arrest, contribute to a global metabolic environment that is not conducive to neurological recovery [8,32,53]. Hepatic and renal dysfunction may lead to accumulation of endogenous toxins, while systemic inflammation may exacerbate cerebral dysfunction through both direct and indirect mechanisms [54,55,56]. These confounding factors may significantly impair the reliability of early neurological assessment and should be carefully considered before prognostic conclusions are made.

#### 3.1.5. Cerebral Perfusion Heterogeneity and Differential Hypoxia

**Figure 1:**
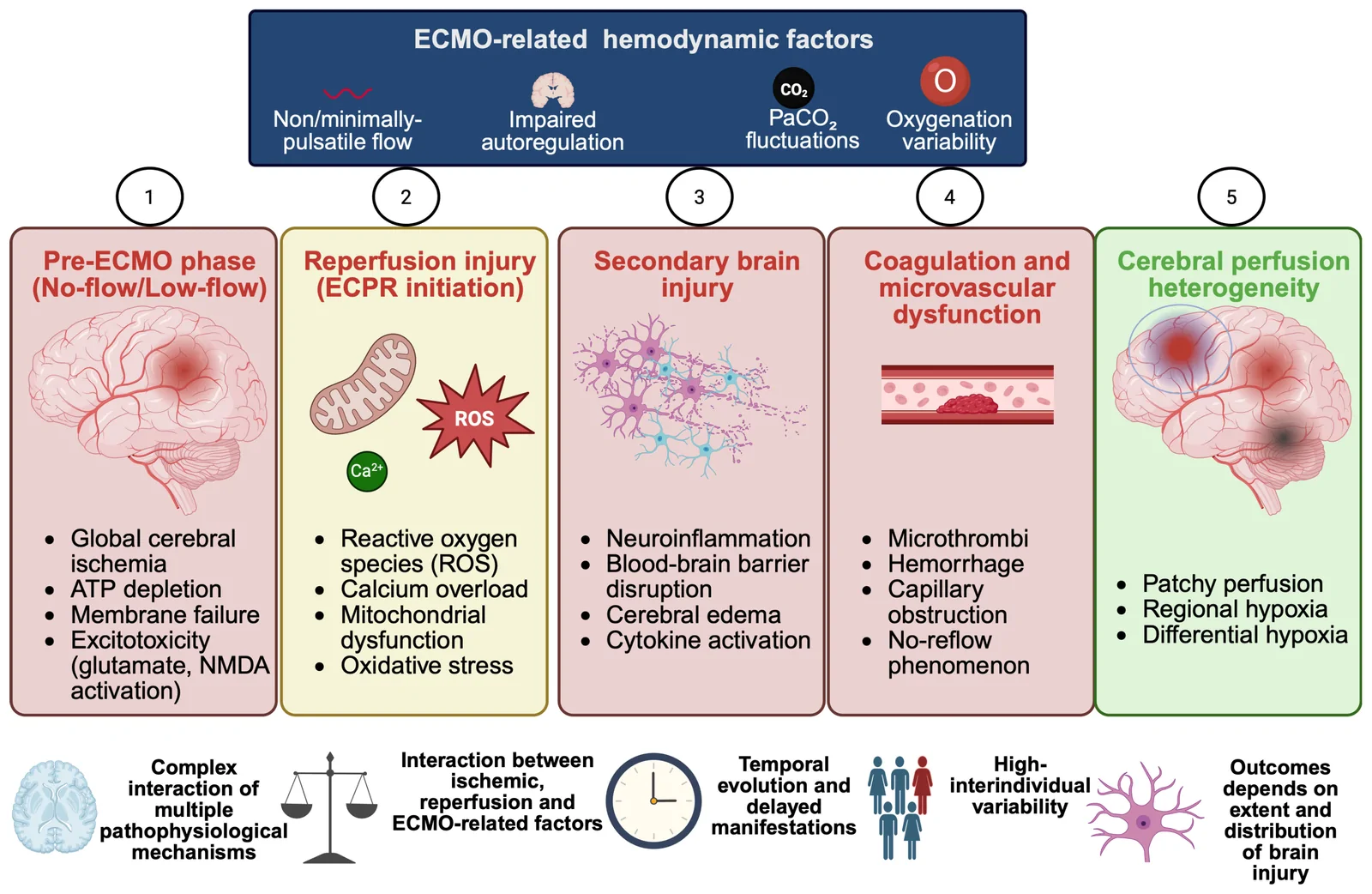
Pathophysiological mechanisms of brain injury in ECPR. Created in BioRender. Pirri, C. (2026). https://BioRender.com/cu7ruju.

Despite restoration of systemic circulation, cerebral perfusion in patients undergoing ECPR remains highly heterogeneous, reflecting a complex dissociation between microcirculatory parameters and macrovascular blood flow [7,57,58] (Figure 1). This mismatch represents a critical and often underrecognized mechanism of ongoing brain injury. At the microcirculatory level, reperfusion is frequently incomplete and spatially uneven. Even in the presence of apparently adequate systemic hemodynamics, regional hypoperfusion may persist due to endothelial dysfunction, capillary obstruction and impaired vasoreactivity [59,60]. This phenomenon, often referred to as “no-reflow phenomenon”, leads to patchy areas of insufficient oxygen delivery coexisting with regions of relative hyperperfusion. As a result, cerebral tissue oxygenation becomes highly variable, promoting localized ischemia and metabolic distress that may not be captured by global monitoring techniques [60]. In addition to microvascular heterogeneity, ECPR introduces unique patterns of regional oxygenation related to interaction between native cardiac output and extracorporeal flow [60]. In peripheral V-A ECMO, the mixing of oxygenated blood from the extracorporeal circuit with blood ejected by the left ventricle creates a dynamic distribution of oxygen content along the aortic axis. When native cardiac function recovers in the presence of persistent pulmonary dysfunction, poorly oxygenated blood may preferentially perfuse the proximal aorta and supra-aortic vessels, including the cerebral circulation, while the lower body receives well-oxygenated blood from the ECMO circuit. This condition, commonly referred to as differential hypoxia or Harlequin syndrome (or dual circulation), exposes the brain to selective hypoxemia despite seemingly adequate systemic oxygenation [36]. The coexistence of microcirculatory dysfunction and regional oxygenation disparities contributes to a highly heterogenous and dynamic pattern of cerebral injury in ECPR patients. From a prognostic standpoint, this spatial variability challenges the interpretation of both clinical and instrumental findings, as global parameters may not accurately reflect regional brain viability [6,8,53].

### 3.2. Timing of Neuroprognostication

**Figure 2:**
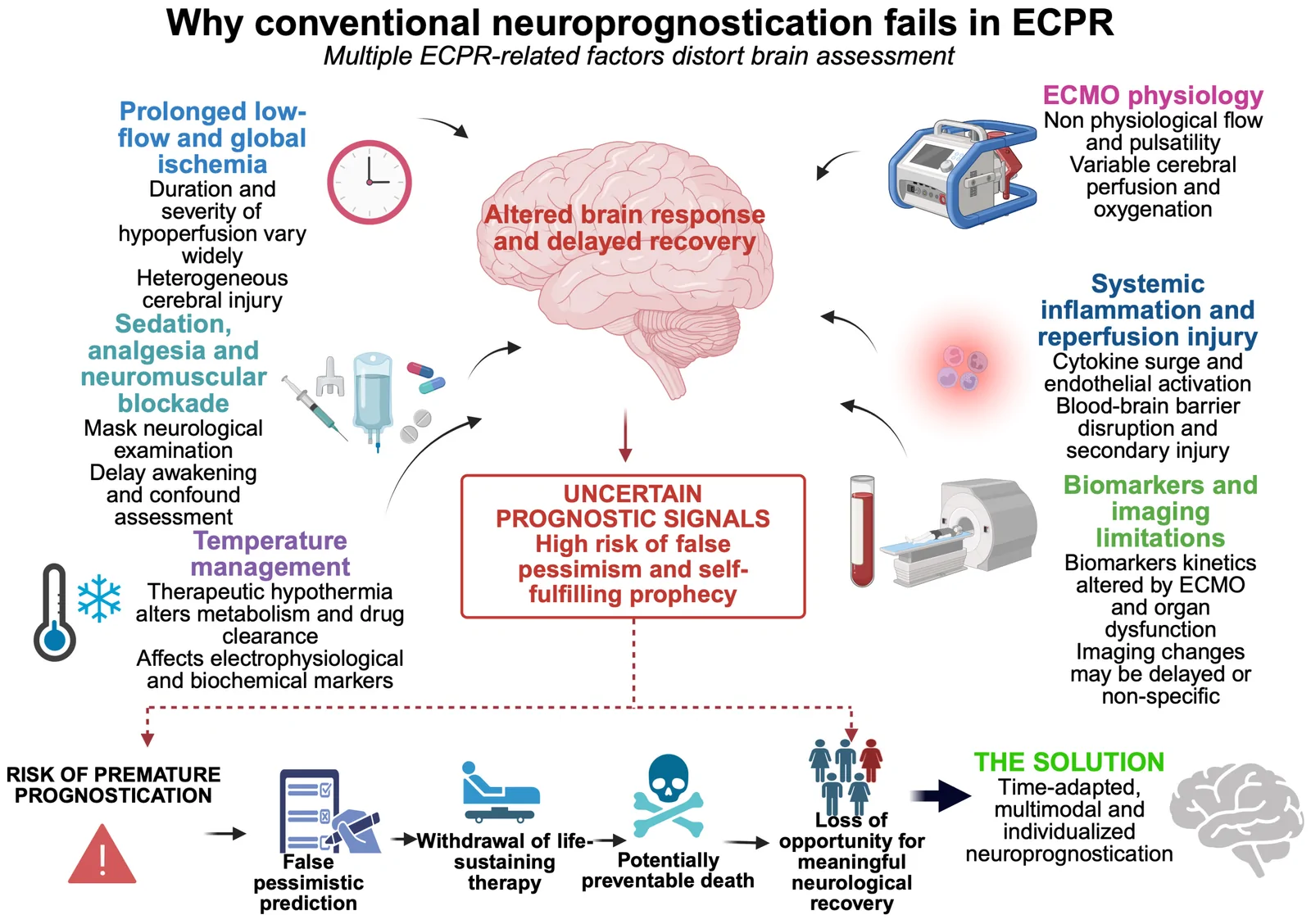
Pathophysiological and temporal determinants of delayed neuroprognostication after ECPR compared with conventional cardiac arrest. Created in BioRender. Pirri, C. (2026) https://BioRender.com/5kjlxfs.

The temporal dimension of neuroprognostication represents one of the most critical and, at the same time, most problematic aspects of post-cardiac arrest care. While current international guidelines for conventional cardiac arrest recommend a structured prognostic assessment at ≥72 h after return of spontaneous circulation (ROSC), the applicability of such timeframes to patients undergoing extracorporeal cardiopulmonary resuscitation (ECPR) remains highly questionable [10,52,53] (Figure 2). In the ECPR population, the concept of a clearly defined “starting point” for neurological recovery is inherently blurred. Unlike conventional resuscitation, where ROSC marks the beginning of reperfusion, ECPR is often preceded by prolonged low-flow states and followed by a phase of extracorporeal-supported circulation that may not immediately restore physiological cerebral perfusion. As a result, the trajectory of brain injury and recovery is temporally shifted and biologically heterogeneous. This temporal heterogeneity is largely explained by the distinct evolution of the pathophysiological mechanisms described in Section 3.1. While no-flow and low-flow injuries determine the initial burden of hypoxic–ischemic damage, several secondary processes continue to evolve after ECMO initiation [5,16,17]. Reperfusion injury unfolds over hours to days through oxidative stress, mitochondrial dysfunction, endothelial activation and blood–brain barrier disruption [17,19,41]. ECMO-related cerebral hemodynamic alterations, including impaired autoregulation, non-pulsatile flow, PaCO2 fluctuations and variable oxygen delivery, may dynamically influence cerebral perfusion throughout the early support period [39]. Neuroinflammatory and coagulation-related mechanisms may further amplify brain injury over subsequent days, contributing to cerebral edema, microvascular dysfunction, ischemic lesions and hemorrhagic complications. At the same time, prolonged sedation, neuromuscular blockade, organ dysfunction and metabolic derangements may suppress neurological responsiveness well beyond the conventional 72 h timeframe. Finally, cerebral perfusion heterogeneity and differential hypoxia may result in evolving and regionally variable patterns of injury [9,19,32,43,44]. Consequently, neurological recovery in ECPR patients may be delayed or obscured by multiple overlapping mechanisms, providing a pathophysiological rationale for a cautious and longitudinal prognostic approach. Several factors contribute to this temporal uncertainty (Table 2) [9,10]. Deep and prolonged sedation is frequently required in patients supported with V-A ECMO, both to ensure ventilatory synchrony and to prevent circuit-related complications. The pharmacokinetics of sedative agents are profoundly altered by critical illness, organ dysfunction and extracorporeal circulation itself, often resulting in delayed drug clearance and prolonged suppression of neurological responsiveness [48,49,50]. Consequently, early clinical examination may be unreliable for extended periods, well beyond the conventional 72 h window. In addition, extracorporeal support introduces hemodynamic conditions that may delay or modulate cerebral recovery. Altered pulsatility, variable cerebral perfusion and ongoing systemic inflammation may contribute to a prolonged phase of secondary brain injury, further complicating the interpretation of early prognostic indicators [7,9,10]. A major implication of this temporal complexity is the heightened risk of premature prognostication. Early withdrawal of life-sustaining therapies (WLSTs), based on incomplete or unreliable neurological assessment, may lead to self-fulfilling prophecies, thereby biasing both individual outcomes and the interpretation of prognostic studies [61,62,63]. This issue is particularly relevant in ECPR cohorts, where decisions are often made in the setting of high-resource utilization and uncertain benefit [64]. Emerging clinical observations suggest that neurological recovery in ECPR-treated patients may be delayed compared with conventional cardiac arrest populations [9]. Cases of meaningful neurological improvement occurring several days, or even weeks, after ECMO initiation have been increasingly reported, especially among younger patients, those with potentially reversible etiologies and those with shorter no-flow durations [9,65,66]. These findings challenge the traditional paradigm of early prognostication and support a more cautious, longitudinal approach. Rather than adhering to rigid temporal thresholds, neuroprognostication in ECPR should be conceptualized as a dynamic and iterative process, integrating evolving clinical, electrophysiological and imaging data over time. In this perspective, time is not merely a passive parameter but an active determinant of diagnostic reliability. Delaying definitive prognostic conclusions until confounding factors have been minimized and multiple modalities have converged toward a consistent interpretation appears to be a more appropriate strategy in this complex population.

### 3.3. Multimodal Neuroprognostication

Neuroprognostication in patients undergoing ECPR requires a multimodal approach integrating clinical, electrophysiological, imaging and biochemical data (Table 3 and Table 4). Given the profound limitations affecting individual prognostic tools in this setting, no single parameter can be considered sufficiently reliable in isolation. Instead, prognostic assessment should rely on the convergence of multiple independent indicators, interpreted within an appropriate temporal framework and in the absence of major confounding factors [6,10]. This integrative strategy is particularly relevant in ECPR-treated patients, in whom altered physiology and delayed neurological recovery significantly reduce the predictive value of conventional markers derived from non-ECMO populations.

The contribution of each prognostic modality is not static but varies according to the temporal phase following ECPR. During the first 24 h, the main objective is the detection of catastrophic brain injury and the identification of major confounding factors. In this phase, brain CT, continuous EEG monitoring and neuromonitoring techniques such as NIRS may provide valuable information, whereas clinical examination is frequently limited by sedation, neuromuscular blockade and metabolic disturbances. Between 24 and 72 h, serial neurological assessments, EEG evolution, neuroimaging findings and biomarker trends may contribute to risk stratification, although prognostic certainty generally remains limited. Beyond 72 h, after major confounders have been minimized, neurological examination progressively regains reliability and should be interpreted together with electrophysiological, imaging and biochemical data. In selected patients, particularly when recovery remains uncertain or delayed, advanced neuroimaging and longitudinal reassessment beyond the first week may provide additional prognostic information [6]. Consequently, multimodal neuroprognostication after ECPR should not be viewed as the simultaneous application of multiple tests but rather as a dynamic process in which the relative diagnostic value of each modality evolves over time (Table 5). The rationale for a multimodal neuroprognostication strategy is also rooted in the multifactorial nature of brain injury after ECPR. No single modality can adequately capture the entire spectrum of hypoxic–ischemic, inflammatory, vascular and metabolic alterations that characterize post-cardiac arrest brain injury [9,10,13]. Instead, each prognostic tool interrogates distinct pathophysiological domains. Clinical examination primarily reflects the integrity of brainstem and cortical function. EEG provides information on functional neuronal activity and cortical network recovery, making it particularly relevant for detecting the consequences of diffuse neuronal dysfunction and ongoing epileptiform activity. Biomarkers such as neuron-specific enolase reflect neuronal injury and cellular breakdown, whereas neuroimaging identifies structural manifestations of brain damage, including cerebral edema, ischemic lesions, hemorrhage and blood–brain barrier disruption. NIRS and other neuromonitoring techniques provide indirect information on cerebral oxygen delivery and perfusion, which may be altered by ECMO-related hemodynamic disturbances [10,67,68,69,70,71,72,73,74].

The integration of these complementary modalities therefore mirrors the biological complexity of ECPR-associated brain injury and represents the conceptual basis of contemporary multimodal neuroprognostication. These temporal variations in diagnostic performance form the rationale for the phase-specific framework proposed in Table 4, in which the relative contribution of each prognostic modality is interpreted according to the evolving biological and clinical context following ECPR. Evidence regarding neurological recovery after ECPR derives from a combination of observational cohorts, randomized controlled trials and large registry analyses. A summary of the neurological outcomes reported in the most relevant ECPR studies and registries is provided in Table 6.

#### 3.3.1. Clinical Examination

Clinical examination remains a fundamental component of neuroprognostication after cardiac arrest; however, its reliability in patients undergoing ECPR is significantly reduced and requires careful interpretation. Assessment of brainstem reflexes, including pupillary, corneal and cough reflexes, represents a cornerstone of neurological evaluation. Among these, the bilateral absence of pupillary light reflex has traditionally been considered a robust predictor of poor neurological outcome in conventional post-cardiac arrest populations [52,53,92]. In the context of ECPR, however, its prognostic value may be attenuated, particularly when assessed early, due to the influence of sedation, altered cerebral perfusion and delayed neuronal recovery [9,10]. Quantitative pupillometry has emerged as a promising tool to enhance the objectivity and reproducibility of pupillary assessment [93]. Parameters such as the Neurological Pupil index provide more reliable information than subjective examination, although their prognostic performance in ECMO-supported patients remains incompletely validated [75]. Motor response to noxious stimuli is another key element of clinical evaluation but is particularly susceptible to confounding in ECPR. The use of sedative and neuromuscular blocking agents, as well as the frequent presence of critical illness-related neuromuscular weakness, may significantly impair motor responsiveness, limiting its interpretability. Importantly, the absence of clinical signs of neurological function in the early phases after ECPR should not be equated with irreversible brain injury. Delayed awakening has been increasingly recognized in this population, and reliance on early clinical findings alone may lead to falsely pessimistic prognostic conclusions [9,10,94]. Overall, while clinical examination remains an essential component of multimodal assessment, its role in ECPR should be considered supportive rather than definitive. Findings must be interpreted cautiously, in conjunction with other modalities and preferably after exclusion of major confounding factors and within an appropriate temporal context.

#### 3.3.2. Electroencephalography

Electroencephalography (EEG) represents a key component of multimodal neuroprognostication after cardiac arrest, providing real-time information on cortical activity and functional brain integrity. In patients undergoing ECPR, EEG retains an important role, although its interpretation requires particular caution due to the complex physiological and pharmacological context. Certain EEG patterns have been consistently associated with poor neurological outcomes in conventional post-cardiac arrest populations. These include highly malignant patterns such as suppressed background activity, burst suppression with identical bursts and unreactive status epilepticus. When observed in appropriate clinical conditions and after exclusion of major confounders, these findings may suggest severe and diffuse cerebral dysfunction [52,53,95,96]. However, the prognostic performance of these patterns in ECPR-treated patients is less well established [67]. The presence of ongoing sedation, altered drug metabolism and non-physiological cerebral perfusion may significantly influence EEG background activity and reactivity [68]. As a result, patterns traditionally considered highly specific for poor outcome may be less reliable when assessed early or in the presence of unresolved confounding factors. Continuous EEG monitoring offers several advantages in this setting. It allows for detection of dynamic changes in cerebral activity over time, identification of subclinical seizures and assessment of background reactivity [69,70]. This temporal dimension is particularly relevant in ECPR, where delayed neurological recovery and evolving brain injury may lead to changes in EEG patterns beyond the initial phases of care. EEG reactivity to external stimuli is generally regarded as a favorable prognostic sign, reflecting preserved cortical responsiveness. Conversely, persistent lack of reactivity has been associated with poor outcomes, although its specificity may be reduced in the presence of sedation or metabolic disturbances. Similarly, the occurrence of electrographic seizures or status epilepticus is associated with worse prognosis, but their interpretation should consider the overall clinical and electrophysiological context [10,52,53,95]. The integration of EEG data with clinical examination, neuroimaging and other monitoring modalities is essential to improving prognostic accuracy. In particular, the concordance of highly malignant EEG patterns with other indicators of severe brain injury may strengthen the reliability of prognostic assessment. Overall, while EEG remains a valuable tool for neurological monitoring in ECPR-treated patients, its prognostic utility is highly dependent on timing, clinical context and the exclusion of confounding factors. A cautious and longitudinal interpretation, preferably based on continuous monitoring, may help support neuroprognostication in this complex population.

#### 3.3.3. Neuroimaging

Neuroimaging plays a central role in the assessment of hypoxic–ischemic brain injury after cardiac arrest, providing structural information that complements clinical and electrophysiological findings [96]. In patients undergoing ECPR, both computed tomography (CT) and magnetic resonance imaging (MRI) are widely used; however, their prognostic interpretation requires careful consideration of timing, technical limitations and ECMO-specific factors [80,81,97,98]. Brain CT is typically the first-line imaging modality due to its wide availability and feasibility in critically ill patients. Early CT findings may include loss of gray–white matter differentiation, sulcal effacement and signs of cerebral edema. Quantitative assessment using the gray-to-white matter ratio (GMR) has been proposed as an objective marker of hypoxic–ischemic injury and has shown prognostic value in conventional post-cardiac arrest populations. Nevertheless, in ECPR-treated patients, early CT may underestimate the extent of brain injury, particularly when performed within the first hours after reperfusion, as structural changes may not yet be fully established [10,52,53,77,92,99]. Magnetic resonance imaging, particularly diffusion-weighted imaging (DWI), is more sensitive for the detection of early ischemic injury and allows for a more accurate characterization of the extent and distribution of cerebral damage. Diffuse cortical and deep gray matter diffusion restriction is generally associated with poor neurological outcomes. However, the timing of MRI acquisition is critical, as both false-negative and evolving findings may occur depending on the phase of injury. In addition, access to MRI in ECMO-supported patients may be limited by logistical challenges, including the need for specialized equipment, transport-related risks and device compatibility [52,53,78,79,80]. Neuroimaging in ECMO-supported patients is further complicated by important logistical and safety considerations. Acute brain injury is common in this population, with observational studies reporting neurological complications in up to 40–50% of patients receiving V-A ECMO. Recent technological advances may help overcome some of these limitations. In particular, the SAFE MRI ECMO study demonstrated the feasibility and safety of bedside ultra-low-field portable magnetic resonance imaging (ULF-pMRI) in critically ill patients receiving ECMO support, potentially expanding access to serial neuroimaging without the risks associated with inter-hospital transport and conventional MRI systems. Although further validation is required, these emerging technologies may facilitate the earlier detection and longitudinal monitoring of neurological injury in ECMO-supported patients [81].

Importantly, neuroimaging findings in ECPR patients may reflect a heterogenous pattern of injury, including both ischemic and hemorrhagic lesions. Intracranial hemorrhage, which may result from anticoagulation, coagulopathy and rapid fluctuations in arterial carbon dioxide tension during ECMO support, represents a frequent and clinically significant complication that must be carefully evaluated [31,100]. The coexistence of ischemic and hemorrhagic injury further complicates prognostic interpretation and may limit the applicability of conventional imaging-based criteria. Another clinical limitation of neuroimaging in this setting is its inability to fully capture functional brain integrity. Structural abnormalities do not always correlate directly with neurological outcome, particularly in the early phases after ECPR, when evolving injury and delayed recovery may lead to dynamic changes over time [6,10]. Serial imaging, when feasible, may provide additional information on the progression of brain injury and improve the reliability of prognostic assessment. Overall, while neuroimaging provides essential insights into the structural substrate of brain injury, its prognostic value in ECPR is strongly influenced by timing, technical feasibility and the heterogenous nature of cerebral damage. A cautious and context-aware interpretation is therefore required to avoid both underestimation and overinterpretation of imaging findings.

#### 3.3.4. Cerebral Oximetry

Cerebral oximetry, based on near-infrared spectroscopy (NIRS), provides a non-invasive and continuous estimate of regional cerebral oxygen saturation (rSO_2_) offering real-time insights into the balance between cerebral oxygen delivery and consumption [96,101]. In patients undergoing ECPR, this technique has attracted increasing interest as a potential tool for monitoring cerebral perfusion and supporting neuroprognostication. One of the main advantages of cerebral oximetry is its ability to detect rapid changes in cerebral oxygenation, particularly during the early phases of extracorporeal support. Decreases in rSO_2_ may reflect impaired cerebral perfusion or oxygen delivery, while increases may indicate improved hemodynamic conditions. In this context, NIRS monitoring may provide valuable information on the adequacy of cerebral oxygenation during ECMO initiation and subsequent management [73,74]. However, several limitations restrict the prognostic applicability of cerebral oximetry in this setting. First, NIRS measurements are influenced by extracranial contamination, variations in tissue composition and probe positioning, which may affect accuracy and reproducibility. Second, rSO_2_ thresholds associated with neurological outcomes have not been consistently validated in ECPR populations [8,102]. Inter-individual variability and the influence of systemic factors, including hemoglobin concentration, arterial oxygen content and carbon dioxide tension, further complicate the interpretation of isolated measurements. For these reasons, trends over time may be more informative than single absolute values. Persistent low rSO_2_ or failure to improve following ECMO initiation may suggest inadequate cerebral oxygen delivery and potentially worse neurological outcome, whereas improving trends may indicate more favorable conditions [73,103,104,105]. Nonetheless, these associations remain observational and should be interpreted with caution. Importantly, cerebral oximetry does not provide direct information on neuronal function or structural brain injury [106]. As such, its role in neuroprognostication is primarily adjunctive, contributing to the overall assessment of cerebral perfusion rather than serving as an independent prognostic marker. Overall, while cerebral oximetry offers a unique window into cerebral oxygenation dynamics during ECPR, its current role in neuroprognostication remains limited. Integration with other monitoring modalities and careful interpretation within the clinical context are essential to avoiding overreliance on this technique in prognostication decision making.

#### 3.3.5. Circulating Biomarkers

Circulating biomarkers of neuronal and glial injury represent a potentially valuable component of multimodal neuroprognostication after cardiac arrest. Among these, neuron-specific enolase (NSE) and S100 calcium-binding protein B (S100B) are the most extensively studied and are commonly used in clinical practice [52,53,107,108]. However, their interpretation in patients undergoing ECPR is particularly challenging and requires careful consideration of ECMO-related factors. NSE, a glycolytic enzyme predominantly found in neurons, is released into the bloodstream following neuronal injury. Elevated serum levels have been associated with poor neurological outcomes in conventional post-cardiac arrest populations, and specific thresholds have been proposed to support prognostic assessment. Similarly, S100B protein, expressed mainly by astrocytes, reflects blood–brain barrier disruption and glial activation and has been explored as an early marker of brain injury [107,108]. In the context of ECPR, however, the reliability of these biomarkers is significantly limited by several confounding mechanisms. Hemolysis, which is relatively common during ECMO support, may artificially increase NSE levels due to its presence in red blood cells, thereby reducing specificity [109,110]. Extracorporeal circulation itself may further influence biomarker kinetics through hemodilution and potential adsorption within the circuit, resulting in unpredictable plasma concentrations. These factors contribute to substantial variability in measured values and limit the applicability of established prognostic cut-offs derived from non-ECMO populations. Another important limitation is the temporal profile of biomarker release. Peak concentrations may occur at different time points depending on the extent of injury and individual patient characteristics and single measurements may therefore be misleading. Serial assessments may provide more meaningful information by capturing dynamic changes over time, although optimal timing and thresholds remain uncertain in ECPR-treated patients [98,99,100]. Emerging biomarkers, such as glial fibrillary acid protein (GFAP) and ubiquitin carboxy-terminal hydrolase L1 (UCH-L1), have shown promise in the evaluation of brain injury, but data in ECMO populations are still limited and insufficient to support routine clinical use [111,112,113,114,115].

## 4. Discussion

Neurological prognostication in patients undergoing ECPR represents one of the most complex and unresolved challenges in contemporary critical care. While substantial progress has been made in conventional post-cardiac arrest populations, the direct application of these paradigms in ECPR-treated patients remains problematic [5,7,8]. The unique pathophysiological features of extracorporeal support, including prolonged low-flow states, non-physiological hemodynamics, systemic inflammation and metabolic disturbances, introduce a level of complexity that fundamentally alters both the trajectory of brain injury and the reliability of established prognostic tools [7]. The growing recognition of delayed neurological recovery in selected patients further reinforces the need to avoid premature conclusions based on early isolated findings [9].

These observations further support the concept that extracorporeal support fundamentally modifies the temporal evolution of neurological recovery and requires a cautious, longitudinal and physiology-informed prognostic approach.

Importantly, the interpretation of multimodal data requires not only integration across techniques but also careful consideration of timing and clinical context. The persistence of confounding factors, particularly in the early phases after ECMO initiation, may lead to falsely pessimistic assessment. In this regard, the concept of “self-fulfilling prophecy” assumes particular relevance in ECPR populations. Early withdrawal of life-sustaining therapies based on uncertain or premature prognostic indicators may directly influence outcomes, thereby introducing bias at both the individual and population levels [62]. Beyond prognostic uncertainty, ECPR raises significant ethical and resource-related considerations. The high intensity of care, combined with uncertain neurological outcomes, places clinicians in a challenging position when balancing the potential for recovery against the risk of prolonged non-beneficial treatment. In this context, transparent communication with families, multidisciplinary decision making and adherence to a cautious and evidence-informed approach are of paramount importance [65]. A particularly challenging and often underrecognized aspect of neurological assessment in ECPR-treated patients concerns the determination of brain death. Furthermore, the determination of brain death during V-A ECMO is complicated by challenges related to apnea testing. Unlike conventional mechanically ventilated patients, carbon dioxide elimination is substantially influenced by sweep gas flow through the oxygenator. Consequently, standard apnea testing protocols cannot be directly applied and often require progressive reduction in sweep gas flow while maintaining adequate oxygenation and hemodynamic stability. Achieving the required hypercapnic stimulus may be technically demanding, and protocol variability remains considerable across centers. These factors may limit the feasibility and reliability of apnea testing and increase the need for ancillary investigations [116,117,118,119].

While brain death represents the most definitive neurological outcome, its diagnosis in the context of extracorporeal support is inherently complex and may be affected by ECMO-related physiological alterations [116]. The use of ancillary tests aimed at demonstrating the absence of cerebral blood flow, including computed tomography angiography, has gained increasing attention in this setting. However, the interpretation of these techniques in patients supported with veno-arterial ECMO is not straightforward [120]. The presence of non-physiological hemodynamics, including continuous or minimally pulsatile flow, altered arterial pressure profiles and the interaction between native cardiac output and extracorporeal circulation, may significantly influence cerebral perfusion patterns and contrast distribution. Under these conditions, contrast opacification of intracranial vessels may be delayed, reduced or regionally inconsistent, potentially leading to false-positive or false-negative interpretations when standard diagnostic criteria are applied. Moreover, the persistence of partial or non-uniform cerebral blood flow does not necessarily imply preserved neuronal viability, further complicating the distinction between irreversible brain injury and residual perfusion. These limitations have important clinical implications, as misinterpretation of cerebral perfusion imaging may lead to inaccurate determination of brain death, with potential consequences for decisions regarding withdrawal of life-sustaining therapies and organ donation. Additional ancillary tests, including transcranial Doppler ultrasonography and radionuclide cerebral perfusion studies, may provide complementary information regarding cerebral blood flow. However, these modalities may also be influenced by ECMO-related alterations in cerebral hemodynamics, reduced pulsatility and heterogeneous perfusion patterns. Consequently, both false-positive and false-negative interpretations remain possible when conventional diagnostic criteria are applied. For this reason, determination of brain death in ECMO-supported patients should rely on careful integration of clinical findings, apnea testing when feasible, ancillary investigations and a comprehensive understanding of the underlying extracorporeal physiology [121]. In light of these challenges, a cautious and multidisciplinary approach to brain death determination in ECMO-supported patients is essential [121,122,123]. The need for standardized protocols and ECMO-specific validation of diagnostic criteria represents an important area for future investigation. Looking forward, several directions may help improve neuroprognostication in ECPR. The integration of multimodal monitoring data using advanced analytical approaches, including machine learning techniques, may enhance the ability to identify reliable prognostic patterns. In addition, further research is needed to validate ECMO-specific thresholds for electrophysiological, imaging and biochemical markers, as well as to better define the optimal timing of assessment. The development of standardized protocols tailored to extracorporeal support populations will be essential to reducing variability in clinical practice and improve comparability across studies. This review has several limitations. First, its narrative design does not allow for formal quantitative synthesis or systematic assessment of study quality. Second, the available evidence remains highly heterogenous, reflecting differences in patient selection, ECMO protocols, the timing of assessment and neuroprognostication strategies across studies. In addition, much of the current knowledge is extrapolated from conventional post-cardiac arrest populations, while prospective ECMO-specific data remain limited. Consequently, several considerations discussed in this review should be interpreted as hypothesis-generating rather than evidence-based recommendations. These limitations underscore the need for dedicated multicenter investigations and standardized neuroprognostication frameworks specifically tailored to ECPR-treated patients. Importantly, the framework proposed in this review should not be interpreted as a validated prognostic algorithm. Given the absence of prospective ECPR-specific neuroprognostication studies, several elements necessarily rely on pathophysiological reasoning, indirect evidence and expert interpretation. Therefore, the framework should be regarded as a conceptual and hypothesis-generating tool intended to support multimodal assessment rather than one providing definitive prognostic recommendations. Overall, neuroprognostication after ECPR should not be interpreted as the application of conventional post-cardiac arrest algorithms to a more technologically advanced population. Rather, extracorporeal support fundamentally modifies the temporal evolution, physiological substrate and interpretability of neurological injury. In this context, early isolated findings may be misleading and potentially contribute to self-fulfilling prognostic trajectories. The challenge is therefore not the identification of a single definitive prognostic marker, but the integration of dynamic, multimodal and time-sensitive information within an ECMO-specific physiological framework. Future progress in the field will likely depend on the development of standardized ECPR-tailored protocols, longitudinal assessment strategies and multimodal models capable of accounting for delayed neurological recovery and extracorporeal-related confounders. Ultimately, neuroprognostication in ECPR should evolve from a static prediction-oriented process to a cautious, adaptive and biologically informed approach aimed at supporting clinically and ethically sound decision making.

### Limitations

This review has several limitations. First, it was conducted as a narrative review and therefore does not provide a formal systematic evidence synthesis, risk-of-bias assessment or evidence grading. Second, the available literature specifically addressing neuroprognostication after ECPR remains limited, heterogeneous and largely derived from observational studies, with many concepts extrapolated from conventional post-cardiac arrest populations. Consequently, several elements of the proposed framework rely on pathophysiological reasoning, indirect evidence and expert interpretation rather than prospective validation. Importantly, the framework proposed in this review should not be considered a validated prognostic algorithm and should not be used as a stand-alone basis for withdrawal of life-sustaining therapy (WLST) decisions. Rather, it should be regarded as a conceptual tool intended to support multimodal assessment and clinical judgment. Future multicenter prospective studies are needed to validate its diagnostic performance, reproducibility and clinical utility in ECPR-treated patients.

## 5. Conclusions

Neuroprognostication after ECPR remains fundamentally distinct from conventional post-cardiac arrest assessment due to the complex interaction of extracorporeal physiology, delayed recovery trajectories and multiple neurological confounders. In this setting, reliable prognostic assessment requires a cautious, longitudinal and multimodal approach integrating clinical, electrophysiological, imaging and biochemical data over time. Avoiding premature prognostic conclusions is essential to minimizing the risk of self-fulfilling prophecies and to supporting clinically and ethically sound decision making.

## 6. Future Directions

Future research in neuroprognostication after ECPR should focus on generating ECMO-specific evidence rather than relying on the extrapolation of conventional post-cardiac arrest paradigms. Prospective multicenter studies are needed to better characterize the temporal evolution of brain injury and to validate the prognostic performance of electrophysiological, imaging and biochemical markers within the unique physiological context of extracorporeal support. Particular attention should be directed toward the development of standardized and time-adapted neuroprognostication protocols capable of integrating serial assessment rather than isolated findings. The incorporation of advanced monitoring technologies, including continuous multimodal neuromonitoring, quantitative EEG analysis and automated imaging interpretation, may improve the detection of dynamic neurological changes during ECMO support. Artificial intelligence and machine learning approaches also represent promising tools for integrating complex longitudinal datasets and identifying prognostic patterns that may not be detectable through conventional single-modality analysis. In parallel, future investigations should address the optimization of cerebral perfusion targets, carbon dioxide management and neuroprotective strategies specifically tailored to ECPR physiology. Finally, the development of standardized approaches for brain death determination and withdrawal of life-sustaining therapy decision making in ECMO-supported patients remains an important unmet need. Advancing the field will therefore require not only technological innovation but also greater integration of neurocritical care, extracorporeal support physiology and ethical decision-making frameworks. Beyond improvements in prognostic assessment, future research should also focus on interventions aimed at promoting neurological recovery after ECPR. Areas of active investigation include optimization of targeted temperature management, individualized cerebral perfusion strategies, multimodal neuromonitoring-guided care, neuroprotective pharmacological approaches and advanced rehabilitation pathways. Emerging technologies such as portable neuroimaging, continuous cerebral autoregulation monitoring and artificial intelligence-assisted prognostic models may further contribute to personalized neurocritical care. Ongoing prospective studies are expected to clarify whether these strategies can improve long-term neurological outcomes in ECPR survivors.

## Figures and Tables

**Table 1:** Determinants of brain injury in ECPR: pathophysiological mechanisms and clinical implications.

Domain	Mechanism	Pathophysiological Features	Clinical Implications
Primary injury	No flow/low flow	Global ischemia, ATP depletion, and excitotoxicity	Strongest predictor of outcome
Reperfusion injury	ROS and calcium overload	Oxidative stress and mitochondrial dysfunction	Delayed neuronal damage
ECMO hemodynamics	Non-pulsatile flow and impaired autoregulation	Pressure-dependent perfusion	Risk of hypo/hyperperfusion
Neuroinflammation	Cytokine cascade	BBB disruption and edema	Secondary injury progression
Coagulation disorders	Thrombosis and bleeding	Microthrombi and hemorrhage	Mixed injury patterns
Sedation/metabolic	Drug accumulation and acidosis	Suppressed neurological responses	False pessimistic prognosis
Differential hypoxia	Mixing zone	Regional hypoxemia	Selective brain hypoxia

ATP: adenosine triphosphate; BBB: blood–brain barrier; ECMO: extracorporeal membrane oxygenation; ROS: reactive oxygen species.

**Table 2:** Major confounders affecting neuroprognostication in ECPR patients.

Category	Specific Factors	Impact on Assessment
Pharmacological	Sedation and NMBAs	Suppress consciousness and motor response
Metabolic	Acidosis and electrolyte imbalance	Alter neuronal function
Hemodynamic	Non-pulsatile flow	Unstable cerebral perfusion
ECMO-related	Hemodilution and circuit sequestration	Alter drug kinetics and biomarkers
Respiratory	PaCO_2_ fluctuations	Affect cerebral blood flow
Organ failure	Renal/hepatic dysfunction	Delayed drug clearance

NMBAs: neuromuscular blocking agents; PaCO_2_: arterial partial pressure of carbon dioxide.

**Table 3:** Multimodal neuroprognostication tools in ECPR: strengths, limitations and interpretation.

Modality	Key Finding	Strengths	Limitations in ECPR
Clinical exam	Brainstem reflexes	Bedside, immediate	Sedation confounding
EEG	Malignant patterns	Functional assessment	Drugs effects
CT	GWR reduction	Available	Early false negatives
MRI (DWI)	Diffuse restriction	High sensitivity	Limited feasibility
NIRS	rSO_2_ trends	Continuous	Poor specificity
Biomarkers	NSE, S100B	Quantitative	Hemolysis, altered kinetics

EEG: electroencephalography; CT: computed tomography; MRI: magnetic resonance imaging; DWI: diffusion-weighted imaging; NIRS: near-infrared spectroscopy; rSO_2_: regional cerebral oxygen saturation; NSE: neuron-specific enolase; S100B: S100 calcium-binding protein B.

**Table 4:** Proposed time-adapted and multimodal framework for neuroprognostication after ECPR, illustrating how the diagnostic contribution of different prognostic modalities evolves according to the temporal progression of ECPR-associated brain injury and recovery.

Time from ECMO Initiation	Clinical Priorities	Suggested Modalities	Interpretation Caveats	Suggested Clinical Approach
0–24 h (ultra-early phase)	Hemodynamic stabilization and correction of hypoxia and metabolic derangements	NIRS (trend monitoring and basic neurological exam (limited))	Profound confounding (sedation, shock, and metabolic imbalance); no *validated* prognostic markers	Avoid any prognostication; focus on optimization of systemic and cerebral perfusion
24–72 h (early phase)	Reduction in confounders and initial neurological assessment	EEG (preferably continuous) and brain CT	Sedation; ECMO-related factors may still significantly alter findings; CT may be falsely reassuring	Interpret findings with extreme caution; do not base decisions on single modality
>72 h (intermediate phase)	Structural multimodal assessment	Clinical exam (after sedation washout), EEG, CT/MRI, and biomarkers (serial)	Delayed awakening common; biomarker thresholds not validated in ECMO	Integrate concordant findings across modalities; repeat assessment over time
>5–7 days (delayed phase)	Re-evaluation of neurological trajectory	MRI (DWI), serial EEG, and longitudinal clinical assessment	Persistent confounders still possible; heterogeneity of recovery trajectories	Consider delayed recovery; avoid premature WLST in absence of consistent poor prognostic indicators
>7–10 days (extended phase)	Advanced prognostic definition	Full multimodal integration	Risk of self-fulfilling prophecy if decisions are made prematurely	Prognostication should rely on robust, concordant and persistent findings across modalities

ECMO: extracorporeal membrane oxygenation; EEG: electroencephalography; CT: computed tomography; MRI: magnetic resonance imaging; DWI: diffusion-weighted imaging; NIRS: near-infrared spectroscopy; WLST: withdrawal of life-sustaining therapy.

**Table 5:** Summary of currently available ECPR-specific evidence supporting major neuroprognostication modalities.

Modality	Main ECPR Studies	Main Findings	Major Limitations
Clinical examination	ECPR observational cohorts [75]	Brainstem reflexes retain value after sedation clearance	Major confounding from sedation, ECMO, and metabolic disturbances
EEG	Ben-Hamouda et al., 2022 [10]	Malignant EEG patterns and absent reactivity associated with poor neurological outcome	Sedation confounding and lack of validated ECPR-specific thresholds
CT	Multiple ECPR cohorts [76,77]	Severe cerebral edema and loss of gray–white differentiation associated with poor outcome	Limited sensitivity in early phase
NSE	Brodska et al., 2024 [11]	Higher NSE levels associated with unfavorable neurological outcome	Hemolysis and ECMO-related kinetic alterations
MRI	SAFE-MRI ECMO and small observational ECPR cohorts [78,79,80,81]	Extensive DWI abnormalities associated with poor outcome	Small sample size, timing variability, and feasibility issues
NIRS	Observational ECMO/ECPR studies [73,74,82]	Feasible continuous bedside monitoring of cerebral oxygenation	Lack of validated prognostic thresholds

ECPR: extracorporeal cardiopulmonary resuscitation; EEG: electroencephalography; NSE: neuron-specific enolase; MRI: magnetic resonance imaging; DWI: diffusion-weighted imaging; NIRS: near-infrared spectroscopy; CT: computed tomography.

**Table 6:** Neurological outcomes reported in major ECPR studies and registries.

Study	Design	Population	Neurological Outcome Measure	Main Findings
ARREST trial [4]	RCT	Refractory OHCA	CPC 1–2 (43%)	Higher favorable neurological survive
Prague OHCA [83]	RCT	Refractory OHCA	CPC 1–2 at 180 days (31.5%)	Numerically higher favorable neurological outcome with the invasive strategy but no statistically significant difference
INCEPTION [3]	RCT	Refractory OHCA	CPC 1–2 at 30 days (20%)	No significant improvement in favorable neurological outcome despite a trend toward benefit in selected patients
Tonna et al. ELSO registry [84]	Registry-based observational study	ECPR	Survival and favorable neurological outcome (CPC 1–2): 17%	Outcome strongly influenced by low-flow duration
Pozzi et al. [85]	Observational cohort study	ECPR	CPC 1–2/neurological recovery: 6–7%	Better selection of patients with CA is essential to ensuring meaningful clinical benefit from ECPR
Spangenberg et al. [86]	Observational cohort study	Refractory cardiac arrest treated with ECPR	CPC 1–2 (31%)	Early ECPR implementation within the “golden hour” is associated with improved survival and favorable neurological outcomes
Dennis et al. [87]	Multicenter observational cohort study	Refractory cardiac arrest treated with ECPR	CPC 1–2 (35%)	Favorable neurological recovery was achieved in selected patients, with outcomes strongly influenced by patient selection and low-flow duration
Ellouze et al. [88]	Observational cohort study	OHCA and IHCA treated with ECPR	CPC 1–2 (23–27%)	Comparable neurological outcomes were observed between OHCA and IHCA patients when treated with ECPR, suggesting that timely ECPR implementation may be more important than arrest location
Mazzeffi et al. [89]	Retrospective observational cohort study	Adult cardiac surgery patients with refractory CA treated with ECPR	CPC 1–2 (26.1%)	ECPR provided meaningful survival and favorable neurological recovery in selected post-cardiotomy patients, supporting its role as a rescue strategy
Blumenstein et al. [90]	Propensity-matched observational study	Observed refractory IHCA treated with ECMO	CPC 1–2 (27%)	ECPR was associated with improved survival and favorable neurological outcomes compared with conventional resuscitation
Peigh et al. [91]	Single-center observational cohort study	IHCA treated with ECPR	CPC 1–2 (30%)	CPR enabled meaningful survival with favorable neurological recovery in selected IHCA patients, supporting its use as a rescue strategy when conventional CPR fails

OHCA: out-of-hospital cardiac arrest; IHCA: intra-hospital cardiac arrest; CA: cardiac arrest; CPC: cerebral performance category.

## Data Availability

No new data were created or analyzed in this study. Data sharing is not applicable to this article.

## Abbreviations

The following abbreviations are used in this manuscript:

ECPR	Extracorporeal cardiopulmonary resuscitation
CPR	Cardiopulmonary resuscitation
OHCA	Out-of-hospital cardiac arrest
IHCA	Intra-hospital cardiac arrest
NSE	Neuron-specific enolase
ECMO	Extracorporeal membrane oxygenation
